# Factors associated with use of psychiatric intensive care and seclusion in adult inpatient mental health services

**DOI:** 10.1017/S2045796016000731

**Published:** 2016-10-20

**Authors:** A. E. Cullen, L. Bowers, M. Khondoker, S. Pettit, E. Achilla, L. Koeser, L. Moylan, J. Baker, A. Quirk, F. Sethi, D. Stewart, P. McCrone, A. D. Tulloch

**Affiliations:** 1 Health Service & Population Research Department, Institute of Psychiatry, Psychology & Neuroscience, King's College London, UK; 2 Department of Applied Health Research, University College London, UK; 3 Molloy College, Rockville Centre, New York, USA; 4 School of Healthcare, University of Leeds, UK; 5 College Centre for Quality Improvement, Royal College of Psychiatrists, UK; 6 South London and Maudsley NHS Foundation Trust, UK; 7 Department of Health Sciences, University of York, UK

**Keywords:** Health service research, inpatient psychiatry, risk factors, treatment allocation, violence/aggression

## Abstract

**Aims.:**

Within acute psychiatric inpatient services, patients exhibiting severely disturbed behaviour can be transferred to a psychiatric intensive care unit (PICU) and/or secluded in order to manage the risks posed to the patient and others. However, whether specific patient groups are more likely to be subjected to these coercive measures is unclear. Using robust methodological and statistical techniques, we aimed to determine the demographic, clinical and behavioural predictors of both PICU and seclusion.

**Methods.:**

Data were extracted from an anonymised database comprising the electronic medical records of patients within a large South London mental health trust. Two cohorts were derived, (1) a PICU cohort comprising all patients transferred from general adult acute wards to a non-forensic PICU ward between April 2008 and April 2013 (*N* = 986) and a randomly selected group of patients admitted to general adult wards within this period who were not transferred to PICU (*N* = 994), and (2) a seclusion cohort comprising all seclusion episodes occurring in non-forensic PICU wards within the study period (*N* = 990) and a randomly selected group of patients treated in these wards who were not secluded (*N* = 1032). Demographic and clinical factors (age, sex, ethnicity, diagnosis, admission status and time since admission) and behavioural precursors (potentially relevant behaviours occurring in the 3 days preceding PICU transfer/seclusion or random sample date) were extracted from electronic medical records. Mixed effects, multivariable logistic regression analyses were performed with all variables included as predictors.

**Results.:**

PICU cases were significantly more likely to be younger in age, have a diagnosis of bipolar disorder and to be held on a formal section compared with patients who were not transferred to PICU; female sex and longer time since admission were associated with lower odds of transfer. With regard to behavioural precursors, the strongest predictors of PICU transfer were incidents of physical aggression towards others or objects and absconding or attempts to abscond. Secluded patients were also more likely to be younger and legally detained relative to non-secluded patients; however, female sex increased the odds of seclusion. Likelihood of seclusion also decreased with time since admission. Seclusion was significantly associated with a range of behavioural precursors with the strongest associations observed for incidents involving restraint or shouting.

**Conclusions.:**

Whilst recent behaviour is an important determinant, patient age, sex, admission status and time since admission also contribute to risk of PICU transfer and seclusion. Alternative, less coercive strategies must meet the needs of patients with these characteristics.

## Introduction

In acute psychiatric inpatient settings, patients exhibiting behaviour or symptoms that are particularly distressing or difficult-to-treat can be subjected to coercive measures. One such intervention is transfer to a specialist, high-intensity ward with higher nurse-to-patient ratios and greater levels of security; in the UK, such wards are referred to as psychiatric intensive care units (PICUs). Another such intervention is seclusion, where the patient is isolated in a locked room and observed at regular intervals. Both PICU transfer and seclusion are often implemented on a compulsory basis and the latter is considered to be unpalatable to some nurses (Olofsson *et al.* 1999) and patients (Mind, 2011). Indeed, many secluded patients experience negative feelings including anger, loneliness, sadness, hopelessness and feeling punished and vulnerable (Van Der Merwe *et al.* 2009). It is perhaps therefore unsurprising that there has been a recent drive to reduce coercive treatments in mental healthcare settings (Molodynski *et al.* 2016). An important step towards achieving this goal is to understand which patients are most likely to be subjected to coercive measures and why.

PICU patients are typically male, young (~30 years), diagnosed with schizophrenia or bipolar disorder, legally detained and (in the UK) more likely to be of black African/Caribbean heritage than non-PICU patients (for a review, see Bowers, 2006). Whilst a recent review found that secluded patients are also likely to be young, legally detained and diagnosed with schizophrenia, bipolar disorder and personality disorder, seclusion was not consistently associated with either patient sex or ethnicity (Van Der Merwe *et al.* 2009). Nationwide studies conducted in the Netherlands (Noorthoorn *et al.* 2015) and Finland (Keski-Valkama *et al.* 2010) indicate that both patients with schizophrenia and substance use disorders are at greater risk of seclusion after adjusting for other demographic/clinical factors. With regard to behavioural precursors, aggressive, disruptive and chaotic behaviour, acute psychosis, absconsion and self-harm are all strongly associated with both PICU and seclusion (Bowers, 2006; Van Der Merwe *et al.* 2009). Consistent with these findings, ‘hurting others’ (Noda *et al.* 2013) and aggressive behaviour prior to admission (Flammer *et al.* 2013) have been reported as common precipitants of seclusion in Japanese and German samples, respectively.

There are several notable limitations with the extant literature. First, many of the previous studies describe the characteristics of people transferred to PICU or placed in seclusion (termed here as *cases*) but do not compare these with people who are not (hereafter referred to as *controls*). Furthermore, with some exceptions [e.g., (Flammer *et al.* 2013)] even those reporting differences between cases and controls typically do not look at the role of patient behaviours. As such, our understanding of the factors contributing to PICU and seclusion use is currently limited. There are two important motivations for pursuing research in this field. First, if we seek to reduce the use of these coercive interventions then we must identify those at greatest risk of receiving them. Second, non-randomised studies seeking to evaluate the effectiveness of PICU and seclusion use in reducing disruptive/aggressive behaviours must account for the differences between treated and untreated groups as such differences will influence the estimation of any treatment effect. To this end, we aimed to determine the demographic, clinical and behavioural characteristics associated with both PICU and seclusion use by means of two separate case-control studies employing multivariable statistical analyses.

## Method

### Data source

Data were extracted from the Clinical Record Interactive Search (CRIS) system operated by the South London and Maudsley (SLaM) NHS Foundation Trust (Stewart *et al.* 2009; Perera *et al.* 2016). This database comprises the anonymised electronic patient records of over 250 000 service users, representing nearly all of those people who have been in contact with SLaM services since 2006. Both structured and free-text data are included, the latter comprising largely correspondence and progress notes. CRIS was approved as a dataset for secondary analysis by the Oxfordshire Research Ethics Committee C (08/H0606/71).

### Identification of PICU cases and controls

**Fig. 1.**
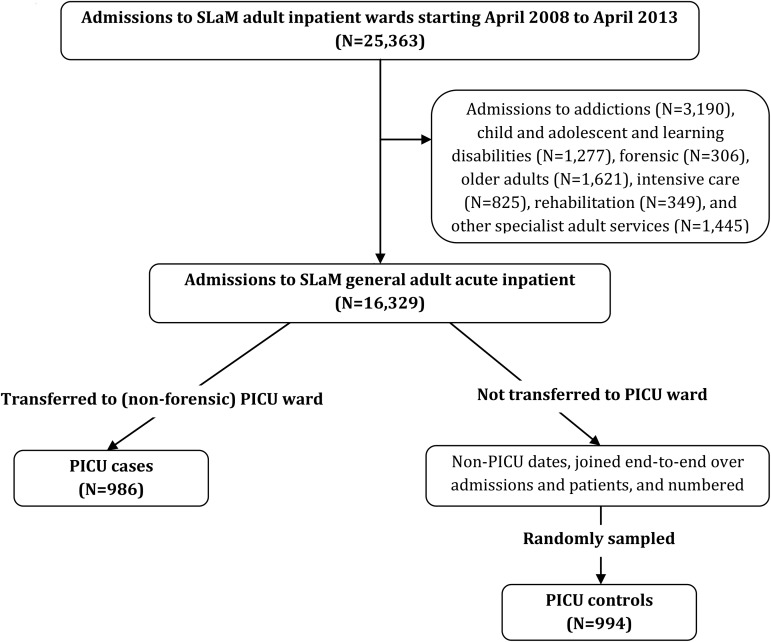
Procedure used to identify psychiatric intensive care unit (PICU) cases and controls.

The procedure used to identify PICU cases and controls is summarised in Fig. 1. We initially created a dataset of all admissions to the five SLaM PICU wards (four general adult and one forensic) between April 2008 and April 2013 using data within structured fields. The following admission types were then excluded: (i) direct admissions to PICU from the community (as this would have required a separate comparison with direct admissions to general wards, for which electronic records would be unavailable), (ii) transfers from forensic wards to PICUs, on the basis that predictors of PICU transfer would differ substantially among general adult and forensic wards (and our primary interest was in the former) and (iii) admissions to the forensic PICU ward. PICU cases (*N* = 986) were defined as patients who were transferred from general adult acute wards to a (non-forensic) PICU. A control group of patients treated in general adult wards was then randomly selected to serve as a comparison for the PICU case group using the following procedure. First, a dataset comprising all admissions to general adult wards within the study period was created and dates corresponding to periods of treatment in PICU wards (including the date of transfer to and out of the PICU ward) were excluded. The resulting non-PICU periods of time were then combined to create a dataset representing all general adult (non-PICU) inpatient days for all patients admitted during the study period; each of these days was then assigned a number such that each corresponded uniquely to a particular date, within a particular admission, for an individual patient. We randomly sampled from this set, fixing the sampling probability such that the number of controls (*N* = 994) would be approximately equal to the number of cases. Fixing both person and date allowed us to code behaviours in the period preceding the sampling date so that for both cases and controls these exposures were defined relative to the date of transfer/non-transfer to PICU.

### Identification of seclusion cases and controls

**Fig. 2.**
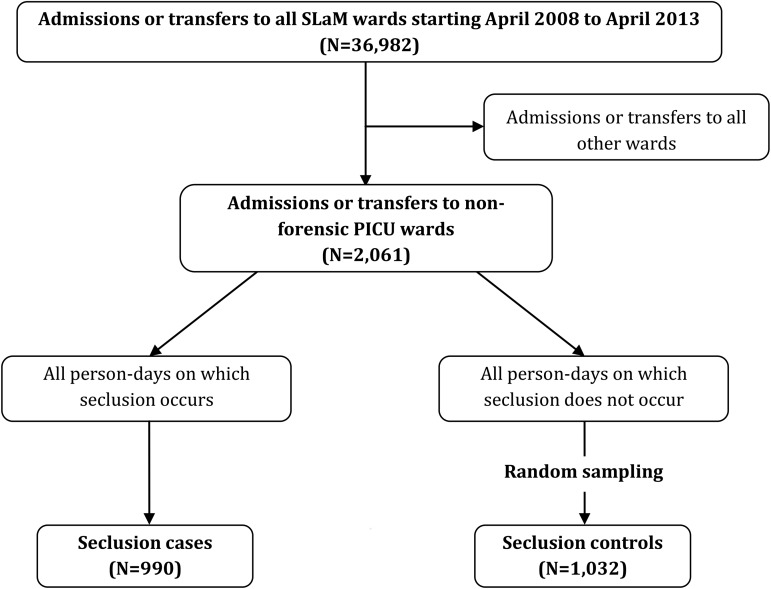
Procedure used to identify seclusion cases and controls.

As detailed in Fig. 2, in order to identify seclusion spells (which are not recorded within structured fields), free-text entries were searched to identify all events containing the words ‘seclusion’, ‘supervised confinement’ and ‘solitary confinement’. These records were then manually cleaned to create a database of all seclusion spells occurring within the study period (*N* = 1478). In order to reduce heterogeneity, seclusions occurring on forensic wards or other non-PICU wards were excluded; thus, only those occurring within the four non-forensic PICU wards (*N* = 990) were examined. Seclusion controls were identified using a similar procedure to that used to identify PICU controls, which involved randomly sampling dates from the set of patient-days on non-forensic PICU wards where the patient was neither in, sent to, nor returned from seclusion. Specifically, we first extracted dates of all non-forensic PICU ward stays occurring within the study period (note, as we did not exclude PICU patients admitted directly from the community, the base population from which potential controls were sampled exceeds the number of cases examined in the PICU analysis) and excluded dates corresponding to days when a patient was in seclusion. These non-seclusion PICU periods were combined and numbered. Random numbers were then generated to identify dates, which corresponded to time periods where a patient was not in seclusion; again, random sampling was used to create a control group (*N* = 1032) of approximately equal size to the seclusion case group.

Cases and controls were not mutually exclusive at the patient level. A single patient may have been transferred to a PICU ward or secluded on multiple occasions and on each occasion would therefore be counted as a PICU or seclusion case, respectively. A single patient might also be included as both a PICU case and a seclusion case. In addition, a PICU or seclusion case could also act as a control in the same analysis, as dates eligible for inclusion in the non-PICU dataset or non-seclusion dataset could be randomly selected as a control period.

### Exposure variables

Data were extracted separately for the PICU and seclusion analyses, but using the same procedure. Sex, ethnicity, date-of-birth and admission date for the current SLaM inpatient episode were extracted directly from structured fields. We calculated age at sampling date (i.e., the date of PICU transfer or seclusion for cases and random sampling date for controls) and the time since admission for the current SLaM inpatient episode, both of which were subsequently recoded into four-level categorical variables. The primary diagnosis recorded closest to the sampling date was extracted from structured fields or retrieved manually from admission, discharge, or tribunal reports if unavailable (<1%). Admission status was determined at midnight on the sampling date and coded as informal, civil section (i.e., formally detained for up to 28 days) and section 3/forensic sections (i.e., formally detained for up to 6 months under a civil section or formally detained under a court/police section).

Putative behavioural exposures were identified from free-text events using a two-stage process described elsewhere (Bowers *et al.* under review). In brief, events recorded on the day of PICU transfer or seclusion commencement, and the 2 days prior to these dates, were first extracted and a subset of these records (pre-PICU: *N* = 500; pre-seclusion: *N* = 500) were reviewed to identify relevant incidents preceding PICU transfer and seclusion (e.g., aggressive, chaotic, or absconding behaviour). The keywords commonly used by clinical staff to describe these behaviours were then recorded. In the second stage, we extracted a random dataset of 350 events (relating to any patient on any admission) that did not occur on the day of PICU transfer/seclusion, or in the 2 days prior to these dates, and used multivariable logistic regression analyses to identify keywords which best discriminated between events occurring prior to PICU transfer/seclusion and random events. Keywords significant at the 0.01 level were used in the final data extraction.

In the final stage, we extracted events occurring on the sampling date and the 2 days prior to these dates that contained the behavioural keywords identified in the aforementioned two-stage process. This process yielded two datasets, a PICU precursor dataset and a seclusion precursor dataset. The PICU precursor dataset included for all PICU cases and controls, all event records occurring within the 3 day time-frame (0, −1, and −2 days prior to sampling date) that contained PICU behavioural terms (22 504 data rows). The seclusion precursor dataset included for seclusion cases and controls, all event records occurring within the 3 day time-frame that contained seclusion behavioural terms (22 239 data rows). Each event record was then manually reviewed to determine whether or not the behaviour(s) implied by the relevant behavioural keyword(s) had actually occurred (keywords and examples of corresponding behaviours are listed in Table 1). Independent ratings were performed by the first and senior author (AEC and ADT) on a subset of free-text records (*N* = 300). Kappa values ranged from 0.66 to 1.00, with 15/18 (83%) behaviours having a Kappa >0.85. Prior to analysis, these coded individual free-text records were combined per case or control, creating a set of variables representing the presence or absence of each behaviour during the entire 3-day time-period prior to the sampling date.

**Table 1.** Keywords used to identify potentially relevant events and examples of corresponding behaviours coded from these events

Keywords	Definition of coded behaviours	Examples of coded behaviours
PICU and seclusion keywords		
Abus*	Verbally abusive behaviour	Patient was abusive; patient began to abuse X; patient was verbally abusive; patient expressed racial abuse
Aggress*	Verbal or physical aggression directed at others or objects	Patient exhibited high levels of aggression; patient was aggressive towards X; patient was verbally aggressive; patient was physically aggressive; patient was behaving aggressively to other patients and staff
Agitat*	Observed agitated behaviour	Patient exhibited agitated behaviour; patient was agitated; medication was given to manage agitation
Demand*	Demanding of resources or change in treatment	Patient was demanding to be discharged/released/taken to the smoking area; patient exhibited demanding behaviour throughout the shift; patient demanded medication/to use telephone/one-to-one
Shout*	Shouting directed at others	Patient shouted at X; patient was overheard shouting at Y; patient shouting to staff to be let out
Threat*	Verbal threats of harm to others	Patient was verbally threatening; patient expressed threats to harm others; patient threatened to become violent
Threw/throw*	Objects thrown at others or destruction of property	Patient threw object at X; patient threw television across room; patient attempted to throw scalding drink at Y
Violen*	Actual physical violence	Patient exhibited violent behaviour; patient was violent; members of staff/patients were subjected to violence
PICU only		
Abscon*	Actual absconsion or serious attempts at absconsion	Patient absconded from hospital; patient forced doors and attempted to abscond; patient continually sought means to abscond from ward throughout shift
Attack*	Actual or attempted attacks	Patient attacked X; patient attempted to attack Y
AWOL	Patient recorded as AWOL	Patient was AWOL from the unit; patient was reported as AWOL to police; patient on leave but went AWOL
Irritable*	Observed irritable behaviour	Patient was irritable throughout shift; patient responded but was irritable
Manic	Observed manic behaviour	Patient presented as manic throughout the night; the patient's behaviour was manic and unmanageable
Refus*	Refusal of staff requests/treatment	Patient refused medication; patient was asked to cooperate but refused; patient refused to return to room
Seclusion only		
Arous*	Observed aroused behaviour	Patient was aroused throughout shift; patient exhibited highly aroused behaviour
Assault*	Actual or attempted physical assault	Patient assaulted X; patient attempted to assault Y; patient was seen to assault Z
Hit*	Actual or attempted physical assault	Patient hit X; patient attempted to hit Y; patient was hitting Z
Restrain*	Restraint of patient by staff	Patient had to be restrained by staff; patient placed in restraint; staff attempted to restrain patient

*Indicates wildcard, used to identify words with 0 or more characters in that position.

### Statistical analyses

Analyses were conducted using Stata version 12. The same procedure was used for the analysis of PICU and seclusion use; in all analyses, the patient ID was included as a random effect in order to account for clustering at the patient-level (i.e., within each dataset, a single patient could represent multiple cases or controls). Mixed effects, multivariable logistic regression analyses were performed to examine associations between all predictor variables and PICU/seclusion status.

## Results

### Predictors of PICU transfer

The PICU cohort comprised 986 cases (PICU transfers) and 944 controls (PICU non-transfers). All these observations originated from 1360 patients, of whom 693 contributed only non-PICU observations, 515 contributed only PICU observations and 152 contributed a mixture. The contribution that each group made to the total number of observations was as follows: those who were never transferred contributed a mean of 1.2 observations (s.d. 0.4); those who were only ever transferred to PICU (in our dataset) contributed a mean of 1.4 observations (s.d. 0.9) and those who were both transferred and not transferred contributed a mean of 3.0 observations (s.d. 1.5).

Multivariable logistic regression analyses (adjusted for all demographic/clinical factors and behavioural precursors, see Table 2) indicated a significant association between patient age and PICU status where the odds of PICU transfer were higher for those aged <25 years (OR = 4.69, *p* <0.001), 25–34 years (OR = 3.94, *p* <0.001) and 35–44 years (OR = 2.11, *p* = 0.009) relative to patients aged ≥45 years. PICU cases were also significantly less likely to be female (OR = 0.37, *p* <0.001); however, there was no association between PICU transfer and patient ethnicity. Patients with a diagnosis of bipolar disorder had significantly greater odds of PICU transfer relative to those with schizophrenia (OR = 2.02, *p* = 0.014), whilst effects for other diagnostic groups were not statistically significant. Patients on a civil section and those on section 3 or a forensic section (predominately sections 37, 35 and 47; which accounted for only a small proportion of sections in this category) were significantly more likely to be transferred to PICU than patients who were informal (OR = 11.62 and 10.75, respectively, *p* <0.001). In addition, the likelihood of PICU transfer decreased as the admission progressed where the odds of transfer were significantly lower for each time period relative to the first 7 days of the admission (*p* <0.05 for all). Several behavioural precursors were also significantly associated with PICU status with small-to-moderate associations observed for ‘Abus*’ (OR = 1.79), ‘Agitat*’ (OR = 3.15), ‘Threat*’ (OR = 3.17), ‘Aggress*’ (OR = 3.58), ‘Violen*’ (OR = 3.79) and ‘AWOL’ (OR = 3.95) (*p* <0.03 for all). Estimates of effect were notably high and for ‘Abscon*’ (OR = 4.68), ‘Threw/Throw*’ (OR = 4.90) and ‘Attack*’ (OR = 28.09) (*p* <0.001 for all).

**Table 2.** PICU cohort: sample characteristics and association with PICU status in multivariable analyses

Risk factor	PICU cases (*n* = 986)	PICU controls (*n* = 994)	OR (95% CI)	*p*
Age: *n* (%)				
<25 years	273 (28)	140 (14)	**4**.**69** (**2.49–8.81)**	**<0**.**001**
25–34 years	342 (35)	216 (22)	**3.94** (**2.19–7.09)**	**<0.001**
35–44 years	220 (22)	273 (27)	**2.11** (**1.21–3.67)**	**0.009**
≥45 years	151 (15)	365 (37) (ref)	–	–
Female sex: *n* (%)	260 (26)	429 (43)	**0.37** (**0.24–0.59)**	**<0.001**
Ethnicity: *n* (%)				
White	273 (28)	423 (43) (ref)	–	–
Black African/Caribbean	625 (63)	465 (47)	1.32 (0.85–2.04)	0.217
Other	88 (9)	106 (11)	1.05 (0.53–2.07)	0.891
Diagnosis: *n* (%)				
Schizophrenia	353 (36)	434 (44) (ref)	–	–
Other psychotic	258 (26)	191 (19)	1.48 (0.91–2.41)	0.114
Bipolar disorder	264 (27)	131 (13)	**2.02** (**1.16–3.52)**	**0.014**
Personality disorder	27 (3)	49 (5)	0.96 (0.27–3.36)	0.951
Other diagnosis	84 (9)	189 (19)	0.80 (0.42–1.54)	0.504
Admission status: *n* (%)				
Informal	37 (4)	445 (45) (ref)	–	–
Civil section	379 (38)	130 (13)	**11.62** (**5.69–23.70)**	**<0.001**
Section 3/forensic	570 (58)	419 (42)	**10.75** (**5.58–20.70)**	**<0.001**
Time since admission: *n* (%)				
≤7 days	409 (41)	191 (19) (ref)	–	–
8–21 days	199 (20)	186 (18)	**0.53** (**0.30–0.91)**	**0.023**
22–60 days	188 (19)	291 (29)	**0.40** (**0.22–0.72)**	**0.002**
>60 days	190 (19)	326 (33)	**0.43** (**0.23–0.79)**	**0.006**
Behavioural precursors: *n* (%)				
Abscon*	143 (15)	14 (1)	**4.75** (**1.88–11.95)**	**0.001**
Abus*	482 (49)	70 (7)	**1.79** (**1.06–3.03)**	**0.030**
Aggress*	602 (61)	58 (6)	**3.58** (**2.11–6.10)**	**<0.001**
Agitat*	670 (68)	140 (14)	**3.15** (**1.96–5.07)**	**<0.001**
Attack*	275 (28)	6 (1)	**28.09** (**9.24–85.41)**	**<0.001**
AWOL*	96 (10)	24 (2)	**3.95** (**1.78–8.75)**	**0.001**
Demand*	456 (46)	114 (12)	1.17 (0.74–1.86)	0.501
Irritable*	529 (54)	144 (14)	1.43 (0.91–2.24)	0.120
Manic	144 (15)	13 (1)	2.67 (0.97–7.35)	0.057
Refus*	773 (78)	418 (42)	0.90 (0.60–1.35)	0.621
Shout*	536 (54)	111 (11)	1.17 (0.72–1.90)	0.538
Threat*	633 (64)	66 (7)	**3.17** (**1.83–5.48)**	**<0.001**
Threw/Throw*	316 (32)	21 (2)	**4.71** (**2.26–9.82)**	**<0.001**
Violen*	199 (20)	5 (1)	**3.79** (**1.15–12.48)**	**0.028**

MHA, Mental Health Act; OR, odds ratio; CI, confidence interval.

*Indicates wildcard, used to identify words with 0 or more characters in that position.

Patient ID included as a random effect in order to account for clustering at the patient level. Results significant at the 0.05 level indicated in bold.

### Predictors of seclusion initiation

The seclusion sample comprised 990 cases (seclusions) and 1032 controls (non-seclusions). All these observations originated from 771 patients, of whom 285 contributed only non-seclusion observations, 203 contributed only seclusion observations and 233 contributed a mixture. In terms of the contribution that each group made to the total number of observations, those who were never secluded, only secluded and both secluded and non-secluded contributed a mean of 1.8 (s.d. 1.5), 1.8 (s.d. 1.4) and 5.0 observations (s.d. 3.7), respectively.

In multivariable logistic regression analyses (Table 3), the likelihood of seclusion was shown to be significantly greater among all other age groups relative to those aged ≥45 years: <25 years (OR = 3.30, *p* <0.001), 25–34 years (OR = 1.92, *p* = 0.017), and 35–44 years (OR = 2.06, *p* = 0.015). Seclusion cases were also significantly more likely to be female (OR = 2.07, *p* = 0.001) but did not differ to the seclusion control group on ethnicity or diagnosis. Relative to informal patients, those on a civil section were significantly more likely to be secluded (OR = 2.29, *p* = 0.022) and the odds of being secluded were increased relative to the first 7 days of the admission for all subsequent time periods (*p* <0.001 for all). Additionally, seclusion status showed significant, small-to-moderate associations with ‘Aggress*’ (OR = 1.85), ‘Arous*’ (OR = 1.87), ‘Hit’ (OR = 1.98), ‘Agitat*’ (OR = 1.99), ‘Violen*’ (OR = 2.09), ‘Assault*’ (OR = 3.31) and ‘Shout*’ (OR = 3.75), and a strong association with ‘Restrain*’ (OR = 6.54) (*p* <0.02 for all).

**Table 3.** Seclusion cohort: sample characteristics and association with seclusion status in multivariable analyses

Risk factor	Seclusion cases (*n* = 990)	Seclusion controls (*n* = 1032)	OR (95% CI)	*p*
Age: *n* (%)				
<25 years	323 (32)	219 (21)	**3**.**30** (**1.85–5.90)**	**<0**.**001**
25–34 years	347 (35)	366 (35)	**1.92** (**1.12–3.28)**	**0.017**
35–44 years	185 (19)	216 (21)	**2.06** (**1.15–3.69)**	**0.015**
≥45 years	135 (14)	231 (22) (ref)	–	–
Female sex: *n* (%)	354 (36)	203 (20)	**2.07** (**1.35–3.17)**	**0.001**
Ethnicity: *n* (%)				
White	199 (20)	253 (24) (ref)	–	–
Black African/Caribbean	690 (70)	657 (64)	1.13 (0.71**–**1.79)	0.609
Other	101 (10)	122 (12)	0.71 (0.34**–**1.46)	0.347
Diagnosis: *n* (%)				
Schizophrenia	323 (33)	435 (42) (ref)	–	–
Other psychotic	273 (28)	262 (25)	0.78 (0.50**–**1.23)	0.287
Bipolar disorder	283 (28)	217 (21)	1.14 (0.70**–**1.86)	0.590
Personality disorder	24 (2)	18 (2)	1.44 (0.42**–**4.94)	0.563
Other diagnosis	87 (9)	100 (10)	1.14 (0.59**–**2.23)	0.696
Admission status: *n* (%)				
Informal	44 (4)	119 (12) (ref)	–	–
Civil section	417 (42)	170 (16)	**2.29** (**1.13–4.68)**	**0.022**
Section 3/forensic	529 (53)	743 (72)	1.34 (0.69**–**2.62)	0.387
Time since admission: *n* (%)				
≤7 days	395 (40)	98 (10) (ref)	–	–
8–21 days	235 (24)	215 (21)	**0.24** (**0.15–0.39)**	**<0.001**
22–60 days	204 (21)	380 (37)	**0.18** (**0.11–0.31)**	**<0.001**
>60 days	150 (16)	339 (33)	**0.26** (**0.15–0.45)**	**<0.001**
Behavioural precursors: *n* (%)				
Abus*	570 (58)	231 (22)	1.37 (0.94**–**1.98)	0.100
Aggress*	654 (66)	192 (19)	**1.85** (**1.30–2.64)**	**0.001**
Agitat*	714 (72)	286 (28)	**1.99** (**1.42–2.78)**	**<0.001**
Arous*	472 (48)	124 (12)	**1.87** (**1.29–2.71)**	**0.001**
Assault*	229 (23)	25 (2)	**3.31** (**1.81–6.06)**	**<0.001**
Demand*	531 (54)	276 (27)	1.24 (0.89**–**1.72)	0.212
Hit*	266 (27)	41 (4)	**1.98** (**1.17–3.35)**	**0.011**
Restrain*	554 (56)	67 (6)	**6.54** (**4.36–9.81)**	**<0.001**
Shout*	573 (58)	217 (21)	**3.75** (**2.58–5.43)**	**<0.001**
Threat*	689 (70)	190 (18)	1.53 (0.98**–**2.37)	0.060
Threw/throw*	328 (33)	77 (7)	1.17 (0.82**–**1.66)	0.385
Violen*	247 (25)	28 (3)	**2.09** (**1.17–3.73)**	**0.012**

MHA, Mental Health Act; OR, odds ratio; CI, confidence interval.

*Indicates wildcard, used to identify words with 0 or more characters in that position.

Patient ID included as a random effect in order to account for clustering at the patient level. Results significant at the 0.05 level indicated in bold.

## Discussion

In this large methodologically robust study, several demographic and clinical factors, including, age, sex, admission status and time since admission, distinguished PICU and seclusion cases from randomly-selected controls. With regard to behavioural precursors, PICU use was most strongly associated with keywords describing incidents of physical aggression and absconsion whilst incidents involving restraint and shouting showed the strongest association with seclusion use.

### Demographic and clinical predictors of treatment

In line with previous studies (Cohen & Khan, 1990; Gordon *et al.* 1998; Brown & Bass, 2004; Stolker *et al.* 2005), younger age was strongly associated with both PICU transfer and seclusion. These findings are unsurprising given that younger age is a well-established risk factor for violence in psychiatric inpatient settings (Cornaggia *et al.* 2011; Dack *et al.* 2013; Iozzino *et al.* 2015). However, the fact that we adjusted for multiple behavioural precursors suggests that this finding is not entirely explained by the fact that younger patients are more likely to be aggressive. This association may reflect some level of bias in clinical decisions (i.e., younger patients being perceived as more risky) or it may be that younger patients differ to older patients on other factors (e.g., frequency or severity of violence) not captured in the current study.

Our finding that male patients were more likely to be transferred to PICU is also consistent with previous work (Hyde *et al.* 1992; Feinstein & Holloway, 2002; Brown & Bass, 2004). Given that we adjusted for behavioural precursors, this sex difference in PICU risk may again be due to unmeasured confounders. Alternatively, this finding may reflect the fact that access to female PICU beds was limited (the trust operated only one female PICU ward, compared with three male wards, at the time of the study). In contrast, females were twice as likely to be secluded as males. Whilst descriptive studies have reported that secluded patients are more commonly male, case-control studies have typically failed to observe such sex differences (Van Der Merwe *et al.* 2009). One possible explanation for our findings may be our use of PICU-based controls. That is, when examined in a PICU population, in which females are under-represented, the increased risk of seclusion among females emerges.

In contrast to recent studies conducted in London and the South East (Feinstein & Holloway, 2002; Brown & Bass, 2004; Pereira *et al.* 2006), patients of black African/Caribbean ethnicity were not significantly more likely to be transferred to PICU compared with white patients. Similarly, patient ethnicity was not associated with seclusion, which conflicts with the findings of the Healthcare Commission investigation in which seclusion rates were higher among ethnic minority groups relative to white British patients (Healthcare Commission, 2005). Interestingly, our unadjusted analyses indicated that individuals of black African/Caribbean ethnicity were three times more likely to be transferred to PICU relative to those of white ethnicity (Bowers *et al.* under review). The fact that the effect of ethnicity on PICU status was greatly attenuated and rendered non-significant in the fully adjusted model is reassuring and suggests a lack of referral bias. That is, whilst black African/Caribbean patients were significantly more likely to be transferred to PICU than white patients, this was fully explained by other risk factors and behavioural precursors.

In contrast to other large studies (Keski-Valkama *et al.* 2010; Noorthoorn *et al.* 2015), patient diagnosis was largely unrelated to either PICU transfer or seclusion with the exception that patients with bipolar disorder were twice as likely to be transferred to PICU compared with patients with schizophrenia. The fact that we adjusted for behaviours and traits commonly associated with this diagnosis (i.e., manic, agitated, demanding and irritable behaviour), implies that bipolar disorder patients may present with other behaviours which cause them to be viewed by clinical staff as needing PICU treatment. Our finding that personality disorders were not over-represented in either PICU or seclusion cases is inconsistent with previous studies (Mattison & Sacks, 1978; Ramchandani *et al.* 1981; Feinstein & Holloway, 2002; Brown & Bass, 2004; Pereira *et al.* 2006) and may be due to the fact that we examined only primary diagnoses listed in clinical files, potentially under-estimating the prevalence of personality disorder (i.e., as comorbid diagnoses will have been missed).

Admission status was strongly associated with PICU use. Only 4% of PICU patients were informal (compared with 45% of non-PICU patients) and indeed, we discovered from discussions with clinical staff that formal detention was generally required on SLaM PICUs. Thus, this very small number of apparently informal patients (as recorded at midnight on the date of transfer) likely reflects administrative delays in updating clinical records. Our finding that patients formally detained were significantly more likely to be secluded is highly consistent with the extant literature (Van Der Merwe *et al.* 2009).

Time since admission was strongly associated with PICU transfer. Whilst this finding is novel, it is not surprising. Patients are often admitted to hospital during a period of acute illness, which then improves following successful treatment; thus, we would expect chaotic/aggressive behaviour to be more prevalent early in the admission. Indeed, a large study of psychiatric inpatients reported that the majority of aggressive incidents occurred within the first 2 days of admission (Barlow *et al.* 2000). However, as we adjusted for behavioural precursors, high levels of aggression upon admission cannot fully account for this finding. Staff might be more inclined to transfer newly-admitted patients, whose propensity for violence is yet unknown, to PICU wards even if they exhibit the same levels of aggression as patients who have been in inpatient care for longer periods. Our finding that the likelihood of seclusion was also higher in the first 7 days of the admission to PICU is consistent with several descriptive studies (Oldham *et al.* 1983; El-Badri & Mellsop, 2002; Kirkpatrick, 1989). Anecdotally, it appeared from the clinical notes that many patients were transferred to PICU in a state of distress/agitation and that seclusion was often initiated as a precaution upon arrival at the PICU. This is consistent with our proposal that this may be a strategy employed by clinical teams to safely manage newly-admitted patients whose level of risk is therefore unclear.

### Behavioural precursors of PICU and seclusion

We identified a number of frequently-occurring keywords in the days preceding PICU transfer and seclusion which described a wide range of patient behaviours (Table 1). Keywords related to difficult-to-manage and verbally aggressive behaviour were particularly common in the 3 days preceding PICU transfer and were observed in 45–78% of PICU cases. Those relating to physically aggressive behaviour were less frequently observed during this time period (20–32% of PICU cases), whilst absconding behaviour was relatively rare (10–15% of PICU cases). Similarly, we identified several keywords related to verbally aggressive and agitated behaviour that were highly prevalent in the period directly preceding seclusion (occurring in 48–72% of seclusion cases) whilst keywords indicating physical aggression were less common (present in 23–33% of cases). Given the wealth of data we examined in the present study, we were not able to characterise the specific behavioural sequences preceding PICU transfer or seclusion; however, the types of behaviours we identified are broadly consistent with previous studies (Bowers *et al.* 2008; Van Der Merwe *et al.* 2009). Interestingly, although we observed a considerable degree of overlap between PICU and seclusion keywords, absconsion keywords were notably absent in the pre-seclusion events. This may relate to the fact that we examined seclusions occurring on a PICU ward (as opposed to a general adult ward) where may be fewer opportunities to abscond.

### Strengths and limitations

This study has several strengths. Our sample sizes were greater than those of previous studies, increasing the precision of the estimates that we obtained, and we combined novel and robust measurement and sampling techniques allowing us to estimate the effect of the time-varying behavioural factors. There were, however, a number of notable limitations. First, the current study was conducted within a single NHS trust, potentially limiting the extent to which our findings can be generalised to other psychiatric hospitals (particularly those outside the UK). Second, whilst we examined a wide range of behavioural precursors, we may have failed to account for some low-frequency behaviours, such as suicide and self-harm. Third, we focused only on patient characteristics as it was beyond the scope of the study to examine environmental factors, which may influence the decision to initiate PICU transfer and seclusion (e.g., number of staff, staff sex, bed numbers). Such environmental factors are particularly important as they are often dynamic (i.e., amenable to change) and therefore offer the opportunity to identify ways by which PICU and seclusion practices might be modified. A fourth limitation relates to our selection of location-based controls. The fact that PICU and seclusion cases were compared with patients admitted to acute wards who were not transferred to PICU and PICU patients who were not secluded, respectively, might explain why our estimates of effect differ somewhat to those of studies comparing PICU/seclusion cases to the entire patient population. Last, there are of course concerns about the accuracy of data extracted from electronic patient records (indeed, seclusion use was not systematically recorded in structured fields within these records), although these would be expected not to have biasing consequences.

### Implications

Our findings have implications for both clinical services and future research. If we seek to reduce coercive interventions in mental health services, it is important to determine which patients are at greatest risk for such interventions using accurate, unbiased clinical data and multivariable models. In the current study, we show that whilst recent behaviour is clearly an important determinant of PICU and seclusion use, patient age, sex, admission status and time since admission also contribute to risk of receiving these measures. Alternative, less coercive strategies must meet the needs of patients with these characteristics. From a research perspective, given that randomised controlled trials evaluating the effectiveness of seclusion and PICU are likely to pose ethical and logistical issues, it is essential to identify factors that distinguish cases and controls as such factors (if not accounted for by randomisation) will need to be appropriately dealt with in statistical models.

## Availability of Data and Materials

The data accessed by CRIS remain within an NHS firewall and governance is provided by a patient-led oversight committee. Data access is possible (albeit subject to these conditions) and interested parties should contact ADT (alex.tulloch@kcl.ac.uk).
